# The complete chloroplast genome of *Styrax dasyanthus* Perkins (Styracaceae)

**DOI:** 10.1080/23802359.2020.1721375

**Published:** 2020-01-31

**Authors:** Xiaogang Xu, Yaoqin Zhang, Lili Tong, Yabo Wang, Chongli Xia

**Affiliations:** a Co-Innovation Center for Sustainable Forestry in Southern China, Key Laboratory of State Forestry and Grassland Administration on Subtropical Forest Biodiversity Conservation, College of Biology and the Environment, Nanjing Forestry University, Nanjing, China;; b State Environmental Protection Scientific Observation and Research Station for Ecology and Environment of Wuyi Mountains, Nanping, China;; c School of Horticulture and Landscape Architecture, Jinling Institute of Technology, Nanjing, China

**Keywords:** *Styrax dasyanthus*, Styracaceae, chloroplast genome sequence, phylogenetic analysis

## Abstract

In this work, next-generation sequencing was applied to measure the complete chloroplast genome of *S. dasyanthus* for the sake of offering valuable genomic information to promote its conservation. The complete chloroplast genome sequence of *S. dasyanthus* was measured as 157,501 bp in size. It contains the typical structure and gene content of angiosperm plastome, which includes a large single-copy (LSC), a small single-copy (SSC), and 2 inverted repeat (IR) regions of 87,130 bp, 18,277 bp, and 26,047 bp, respectively. The *S. dasyanthus* chloroplast genome has a total of 130 genes including 85 protein-coding genes, 37 tRNA genes, and 8 rRNA genes. Overall, GC contents of the genome were 36.96%. Phylogenetic analysis based on the 29 chloroplast genome measurement demonstrated that *S. dasyanthus* is most closely related to *Styrax grandiflours* Griff.

*Styrax dasyanthus* is widely distributed across central and southern China, which has the value of medicinal, aromatic, ornamental, and timber resources. Comparison of complete plastome sequences further provides the opportunity to explore sequence variation and molecular evolutionary patterns associated with gene loss, rearrangements, duplication, and transfer events (Walker et al. 2014; Weng et al. 2014). But to date, there is still only a few complete cp genome that was characterized for the species in *Styrax*. Here, we developed chloroplast genome sequence of *S. dasyanthus* to further apply developing authentication as well as in distinguishing from its confusing species.

The fresh leaves of *S. dasyanthus* natively habitated in Jinniushan (N32.473, E118.994, Alt. 176 m) in Luhe District, Nanjing, China, were collected for whole-genome DNA extraction. The voucher specimen was deposited in the herbarium of Nanjing Forestry University (accession number: NF2018097). In this study, we determined the complete chloroplast DNA sequence of *S. dasyanthus* by using next-generation sequencing technology. The total DNA extracting and the whole-genome sequencing were conducted by Nanjing Genepioneer Biotechnologies Inc. (Nanjing, China) with the Illumina Hiseq 2500 Sequencing System. The raw reads were filtered by CLC Genomics Workbench v9 and filtered sequences were assembled using the program SPAdes assembler v3.10.1(Bankevich et al. 2012). Finally, the cp genome annotation and visualization were performed using web server CpGAVAS (Liu et al. 2012). The *S. dasyanthus* cp genome was deposited in GenBank (GeneBank accession number: MN329776).

The complete cp genome size of *S. dasyanthus* was 157,501 bp, which was composed of a large single-copy region (LSC) of 87,130 bp, a small single-copy region (SSC) of 18,277 bp, and 2 inverted repeat regions of 26,047 bp. In total, the genome contained 130 genes, including 85 protein-codon genes (79 PCG species), 37 tRNA genes (27 tRNA species), and 8 rRNA genes (4 rRNA species). Among them, 17 genes occur in double copies, including 6 protein-codon genes (*ndhB, rpl2, rpl23, rps7, ycf15,* and *ycf2*), 7 tRNA genes (*tRNA-ACG, tRNA-CAA, tRNA-CAU, tRNA-GAC, tRNA-GUU, tRNA-UGC,* and *tRNA-UUC)*, and 4 rRNA genes (*rrn16, rrn23, rrn4.5,* and *rrn5*), and most of the gene occur as a single copy. Ten genes (*ndhB, rpl2, rpoC1, rps16, tRNA-AAA, tRNA-CGA, tRNA-UAA, tRNA-UGC, tRNA-UUC, tRNA-UUU*) contained 1 intron and 1 gene (*clpP*) contained 2 introns. The whole chloroplast genome GC content is 36.96%, while the value of the LSC, SSC, and IR regions are 34.80, 30.26, and 42.93%, respectively.

In order to reveal the phylogenetic position of *S. dasyanthus* with other members of Styracaceae, a phylogenetic analysis was performed based on 18 complete chloroplast genomes of Styracaceae,and 5 taxa (Symplocaceae, Theaceae, Clethraceae, Actinidiaceae, and Ericaceae) as outgroups. They were all downloaded from NCBI GenBank. The sequences were aligned by MAFFT v7.307 (Katoh and Standley 2013) and the phylogenetic tree was constructed by Figtree v1.4.4. As shown in Figure 1, *S. dasyanthus* is the sister to *Styrax grandiflours* and clustered within the group consisting of the genus that belongs to *Styrax*.

**Figure 1.**
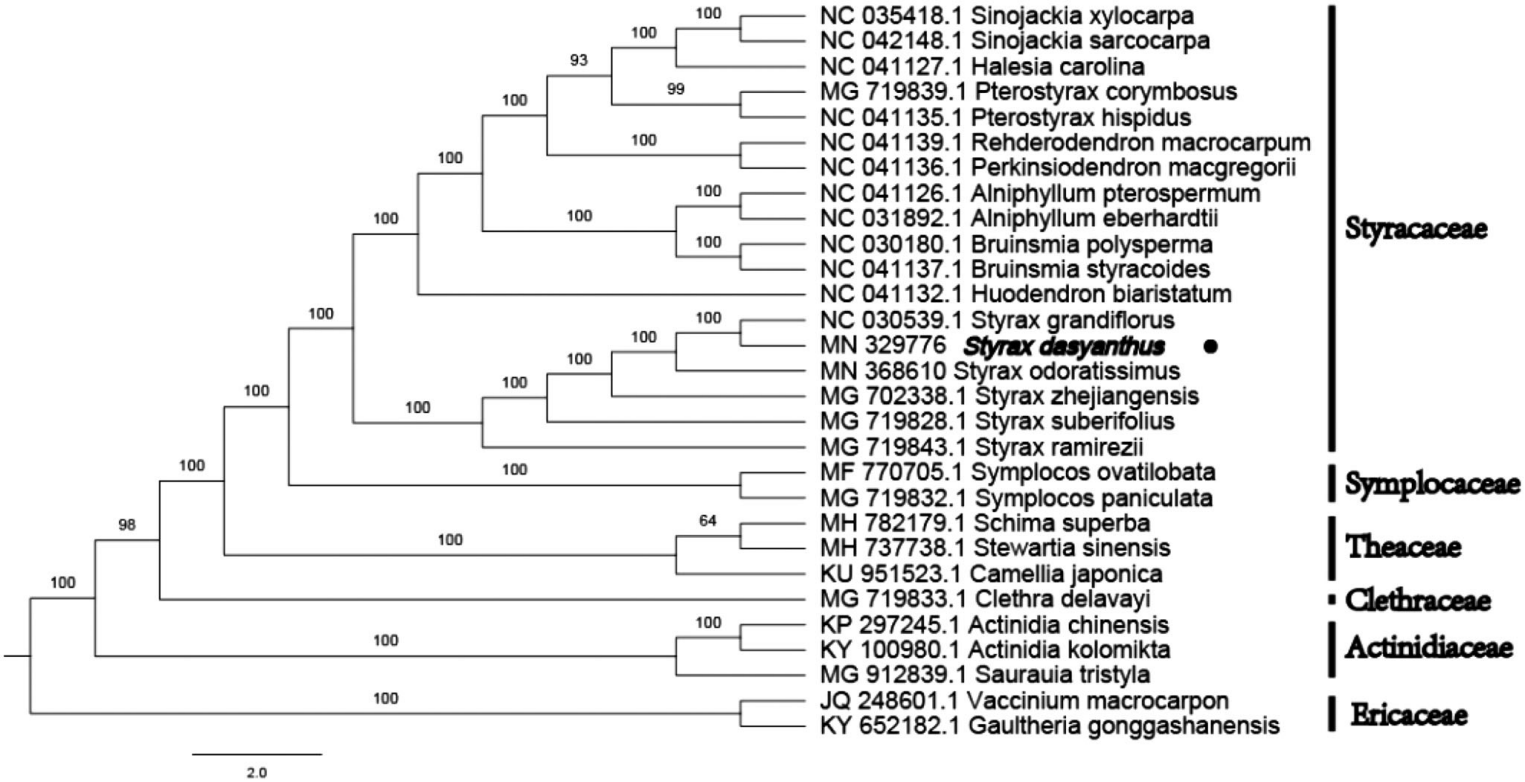
Phylogenetic tree inferred by maximum-likelihood (ML) method based on the complete chloroplast genome of 29 representative species. The bootstrap support values are shown at the branches.
